# Circulating amino acid signature features urea cycle alterations associated with coronary artery disease

**DOI:** 10.1038/s41598-024-76835-7

**Published:** 2024-10-28

**Authors:** Luisa Prechtl, Justin Carrard, Hector Gallart-Ayala, Rébecca Borreggine, Tony Teav, Karsten Königstein, Jonathan Wagner, Raphael Knaier, Denis Infanger, Lukas Streese, Timo Hinrichs, Henner Hanssen, Julijana Ivanisevic, Arno Schmidt-Trucksäss

**Affiliations:** 1 School of Cardiovascular and Metabolic Health, University of Glasgow, 126 University Place, Glasgow, G12 8TA Scotland; 2 Division of Sports and Exercise Medicine, Department of Sport, Exercise and Health, University of Basel, Grosse Allee 6, 4052 Basel, Switzerland; 3 Metabolomics Platform, Faculty of Biology and Medicine, University of Lausanne, Quartier UNIL-CHUV-Rue du Bugnon 19, 1005 Lausanne, Switzerland

**Keywords:** Amino acids, Metabolic signature, Metabolic profiling, Urea cycle, Coronary artery disease, Biomarkers, Cardiology, Cardiovascular biology, Molecular medicine, Metabolomics

## Abstract

Coronary artery disease (CAD) remains a leading cause of death worldwide and imposes a substantial socioeconomic burden on healthcare. Improving risk stratification in clinical practice could help to combat this burden. As amino acids are biologically active metabolites whose involvement in CAD remains largely unknown, this study investigated associations between circulating amino acid levels and CAD phenotypes. A high-coverage quantitative liquid chromatography-mass spectrometry approach was applied to acquire the serum amino acids profile of age- and sex-coarsened-matched patients with CAD (n = 46, 66.9 years, 74.7% male) and healthy individuals (n = 120, 67.4 years, 74.7% male) from the COmPLETE study. Multiple linear regressions were performed to investigate associations between amino acid levels and (a) the health status (CAD vs. healthy), (b) the number of affected coronary arteries, or (c) the left ventricular ejection fraction. Regressions were adjusted for age, sex, daily physical activity, sampling, and fasting time. Urea cycle amino acids (ornithine, citrulline, homocitrulline, aspartate, and arginine) were significantly and negatively associated with CAD, the number of affected coronary arteries, and the left ventricular ejection fraction. Lysine, histidine, and the glutamine/glutamate ratio were also significantly and negatively associated with the CAD phenotypes. Overall, patients with CAD displayed lower levels of urea cycle amino acids, highlighting a potential role for urea cycle amino acid profiling in cardiovascular risk stratification.

Trial registration

The study was registered on https://www.clinicaltrials.gov (NCT03986892) on June 5, 2019.

## Introduction

Coronary artery disease (CAD) remains the leading cause of death worldwide and a significant contributor to disability^1^. Improving prevention and early detection of CAD could mitigate this socioeconomic burden^2^. In clinical practice, biochemical stratification of patients with or at risk of CAD still mainly relies on the measurement of glycated haemoglobin (HbA1c), low-density lipoprotein cholesterol (LDL-C), high-density lipoprotein cholesterol (HDL-C), and triglycerides^3^. These biomarkers, however, represent only partly the metabolic disturbances occurring with CAD^4^. Advances in mass spectrometry and bioinformatics recently enabled the acquisition of multiparametric metabolic profiles, including quantifying a wide range of circulating metabolites, which could improve cardiovascular risk stratification^5^.

While lipids have long been used as biomarkers of CAD, amino acids have sparsely been investigated in this context and are traditionally considered as building blocks of proteins. However, amino acids act as signalling molecules to regulate metabolic homeostasis and are important precursors to hormones and neurotransmitters^6^. Furthermore, amino acid and lipid metabolisms are closely related^7^. Indeed, amino acid catabolism produces acetyl-coenzyme A, a substrate of fatty acid synthesis, linking elevated amino acid concentrations to increased levels of circulating fatty acids and acylcarnitines^8^.

So far, methionine, histidine, glutamine, branched-chain amino acids, phenylalanine, and citrulline have been positively associated with the risk of incident cardiovascular events^5,9,10^. At the same time, homoarginine and tryptophan are believed to be atheroprotective^11^. Interestingly, arginine has been positively and negatively associated with CAD^5^. Building upon these promising findings, this study aimed to acquire the circulating amino acid profile of patients with CAD and age- and sex-coarsened-matched healthy individuals from the COmPLETE study^12^. Further, associations between circulating amino acid levels and CAD phenotypes were investigated. To acquire the most comprehensive panel possible of circulating amino acids, a novel high-coverage targeted metabolomics approach was applied^7^.

## Materials and methods

### Study design and participants

Subsets of the COmPLETE Heart (n = 46, 91.3% male) and COmPLETE Health (n = 183, 61.2% male) samples were investigated. The COmPLETE Heart subset comprised solely patients diagnosed with CAD, as verified by senior cardiologists. For this study, CAD was defined as "a pathological process characterised by atherosclerotic plaque accumulation in the epicardial arteries"^13^. Patients were included based on the diagnosis in the medical reports provided by treating cardiologists. In the case of clinically significant concomitant disease states (i.e. non-cardiovascular diseases), patients were excluded from the COmPLETE Heart study. As the aim was to investigate associations between CAD diagnosis, as evidence of a systematic atherosclerosis cardiovascular disease, and circulating amino acids, no difference was made between patients with or without stenting of coronary arteries and with or without left main coronary artery disease. As previously reported in the study protocol, "patients with unstable angina pectoris, uncontrolled brady- or tachyarrhythmia, permanent atrial fibrillation, severe valvular disease, acute myocardial infarction, transient ischemic attack, or stroke in the last three months were excluded"^12,14^. "Clinically healthy participants from the Basel area (Switzerland) who had no exercise-limiting chronic diseases and were non-smokers (or had quit at least ten years previously) were included in the COmPLETE Health sample"^12,14^. "This excluded participants with a history of CAD, stroke, heart failure, lower-extremity artery disease, any malignant tumour, diabetes, obesity, clinically apparent kidney failure, severe liver disease, chronic obstructive pulmonary disease (GOLD stadium II-IV), arterial hypertension (grades II-III according to the classification of the European Society of Cardiology), drug or alcohol abuse, exercise-limiting osteoporosis or musculoskeletal conditions and clinically manifest Alzheimer’s disease or dementia"^12,14^. The COmPLETE study conformed to the ethical guidelines of the Declaration of Helsinki and was approved by the Ethics Committee of North-Western and Central Switzerland (EKNZ 2017-01451). The study was registered on https://www.clinicaltrials.gov (NCT03986892) on June 5, 2019. All participants provided written informed consent.

### Data collection

All clinical data were gathered between January 2018 and December 2019 at the Department of Sport, Exercise, and Health, University of Basel. The methodology for participant recruitment and data acquisition was detailed within the study protocol^12^. Briefly, participants received instructions to maintain their usual dietary habits before clinical assessments (for the preceding 72 h), refrain from exercise and alcohol consumption (for the past 24 h), as well as abstain from caffeinated beverages (for at least the last 4 h) and food intake (for a minimum of 3 h). Participants were assigned to one of five available time slots (08:00, 10:00, 12:00, 14:00, and 16:00) based on their scheduling availability, with each assessment session lasting approximately 4 h.

Before the clinical examination, participants’ smoking status was evaluated via telephone. Physicians reviewed medical histories and current medications using an on-site questionnaire. Body fat composition was assessed through a four-segment bioelectrical impedance analysis (Inbody 720, Inbody Co. Ltd., Seoul, Korea). Trained physicians conducted transthoracic echocardiography in accordance with international standards^15^. Apical 2- and 4-chamber views were acquired using the Fukuda UF 760 ultrasound scanner (Fukuda Denshi, Tokyo, Japan) equipped with an SA16 (2–5 MHz) transducer (Fukuda Denshi, Tokyo, Japan). A trained cardiologist visually estimated left ventricular ejection fraction (LVEF) using the eyeballing method, demonstrating comparable accuracy to formal echocardiographic techniques for LVEF assessment^16^. The number of affected coronary arteries was determined based on patients’ most recent medical reports. Following 1 h of rest, blood samples were collected from participants in a fasting state (minimum of 3 h) via venipuncture of the cubital fossa (2 × 7.5 mL serum-gel, Monovette^®^, Sarstedt, Nümbrecht, Germany). Serum samples underwent gentle agitation for 30 min, followed by centrifugation (3000 rpm; 10 min; 20–23 °C), aliquoting, and storage at − 80 °C.

Physical activity levels were tracked for 14 days after the clinical assessment using a wrist-worn triaxial accelerometer (GeneActive Activinsights Ltd., Kimbolton, UK). Data analysis was conducted using the validated open-source Excel macro file "General physical activity" (version 2), with total and moderate-to-vigorous physical activity quantified in minutes per day (moderate defined as 4.00–6.99 Metabolic Equivalent of Task (METS) and vigorous as ≥ 7 METS)^17^.

### Standard biochemical analysis

Serum concentrations of total cholesterol, LDL-C, HDL-C, and triglycerides were analyzed using an Olympus AU680 automatic analyzer (Beckman Coulter, Brea, CA, USA), enzymatic reagents (DiaSys, Holzheim, Germany), and secondary standards (Roche Diagnostics, Mannheim, Germany). HbA1c levels were quantified from whole blood through high-pressure liquid chromatography employing D-10 (Bio-Rad, Hercules, CA, USA).

### Sample preparation

To quantify amino acid concentrations, 20 µL of blood serum was extracted using 250 µL of ice-cold methanol spiked with corresponding stable isotope-labelled standards. Subsequently, the solution was adjusted to 300 µL with 0.1% formic acid in water. After vortexing and centrifugation for 15 min at 4 °C and 2700*g*, the supernatants were transferred to liquid chromatography-mass spectrometry vials before injection.

### Amino acid quantification

Serum extracts underwent analysis utilizing hydrophilic interaction liquid chromatography coupled to high-resolution mass spectrometry (HILIC-HRMS) employing a Vanquish Horizon (Thermo Fisher Scientific) ultra-high performance liquid chromatography (UHPLC) system connected to a Q-Exactive™ Focus mass spectrometer interfaced with a heated electrospray ionization (HESI) source operating in positive mode^7^. Chromatographic separation utilized an Acquity BEH amide (1.7 μm, 100 mm × 2.1 mm I.D.) column (Waters, Massachusetts, US). The mobile phase consisted of A = 20 mM ammonium formate and 0.1% formic acid in water and B = 0.1% formic acid in acetonitrile. The gradient elution commenced at 95% B (0–2 min), decreased to 65% B (2–14 min), reached 50% B at 16 min, and was followed by an isocratic step (16–18 min) before a 4-min post-run for column re-equilibration. The flow rate was set at 400 μl/min, the column temperature at 25 °C, and the sample injection volume at 2 μl. The HESI source conditions in positive mode were as follows: sheath gas flow at 60, auxiliary gas flow rate at 20, sweep gas flow rate at 2, spray voltage at + 3 kV, capillary temperature at 280 °C, s-lens radio frequency level at 60, and auxiliary gas heater temperature at 300 °C. Full scan high-resolution mass spectrometry acquisition mode (m/z 50–750) was employed with parameters including a resolution of 70,000 full-width half maximum (FWHM), 1 µscan, 1e6 automatic gain control, and a maximum inject time of 100 ms.

### Data processing

Xcalibur 4.1 (Thermo Fisher Scientific) was used to process raw data files. The peak area of each amino acid was auto-integrated and manually corrected if necessary. Calibration curves and the stable isotope spike (or internal standard spike) at known concentration were used to determine the concentration of each amino acid concentration. A nine-point calibration curve was used to assess the linearity of the mass spectroscopy response of each amino acid. A human plasma standard reference material (Certificate of Analysis, NIST 1950) was evaluated within each batch of samples as an external quality control of the measurement accuracy.

### Statistical analysis

Patients with CAD were age- and sex-coarsened-matched with clinically healthy individuals using the coarsened exact matching and average treatment effect (MatchIt R package, version 4.5.0)^18^. The coarsened exact matching method coarsened covariates into bins before applying exact matching. The balance ultimately comes from weighting the matched observations in the analysis. Thus, the benefit of coarsened exact matching over exact matching is that fewer participants are discarded while balancing between groups is maintained (even if it results in differences in group sizes)^19^.

Three multiple linear regressions were run to exploratively assess associations between circulating amino acids and CAD phenotypes. In addition to testing for associations between amino acids and the presence of CAD, associations with CAD severity were investigated. To this end, the number of affected coronary arteries, as determined by patients’ most recent medical reports, was used as a surrogate of CAD severity^20–23^. Finally, associations between amino acids and LVEF were investigated. LVEF is a functional parameter which has been reported to be associated with mortality and cardiovascular events in CAD patients^24^ and with subclinical atherosclerotic disease burden^25^. To determine for which variables regressions needed to be adjusted, a directed acyclic graph (DAG) was drawn (Supplementary Fig. 1)^26^. Amino acids served as dependent variables, whereas the variables of interest, age, sex, daily total physical activity, sampling time, and fasting time served as independent variables. The variable of interest was a binary variable CAD vs. healthy in the first regression, the number of affected coronary arteries in the second (0, 1, 2, or 3), and the LVEF in the third regression^20^. According to the current heart failure guidelines of the European Society of Cardiology, LVEF was divided into preserved (≥ 50%), mildly reduced (41–49%), or reduced (≤ 40%)^27^. During the review process, the authors were recommended to add for each set of regressions an additional model, in which participants with kidney failure (defined as an estimated glomerular filtration rate, abbreviated eGFR, lower than 60 ml/min/1.73 m^2^) were excluded, and eGFR was used as an additional variable for which regressions were adjusted^28^. eGFR was calculated with the CKD EPI formula using the ckd_epi function of the R package transplantr^29^. To estimate the effects (β coefficient) and the uncertainty of the effects (standard errors, 95% confidence intervals (CI), and p-values), the cluster-robust standard errors method was applied as recommended following matching^30,31^. This method accounts for dependence between observations within matched pairs (i.e. clusters)^30,31^.

Amino acid concentrations were log_2_-transformed before statistical analysis, and continuous dependent and independent variables were z-standardised^32^. Model assumptions were assessed graphically using residual plots. If the proportion of missing data remained below 5%, a complete case analysis was done. Otherwise, multiple imputations would have been considered. All p-values were adjusted using the Benjamini–Hochberg (BH) method^33^. The statistical analyses and all graphs were done with the software R (version 4.1.1)^34^. R-codes are available at https://github.com/JustinCarrard/Amino_acid_profiling_of_CAD_patients. The raw data are available in the Supplementary Table 1.

## Results

### Participants characteristics

Participants’ characteristics are displayed in Table 1. One hundred twenty healthy participants (67.5 years (standard deviation 14.0 years), 68.3% male) could be age- and sex-coarsened-matched to 46 patients with CAD (65.6 (14.0) years, 91.3% male). The coarsened exact matching led to a balanced weighted distribution of age (mean of 67.2 years in the healthy participants vs. 66.9 years in CAD patients) and sex in both groups (74.7% of males in both groups). The healthy individuals showed a mean BMI within the normal range, while CAD patients were overweight on average. According to the European Society of Cardiology classification, mean systolic and diastolic blood pressure values were within the normal (healthy individuals and males with CAD) and high normal range (females with CAD)^35^. All CAD patients and 20.8% of the healthy individuals took antihypertensive drugs. Two-thirds of the healthy population never smoked, 15.6% of CAD patients quit less than ten years ago, and 13.0% were still smoking. As to cholesterol, all the CAD patients except one were taking lipid-lowering drugs (compared to 7.5% of healthy individuals). Thus, healthy individuals exhibited higher mean total cholesterol (5.8 (1.2) mmol/l) and LDL-C (3.2 (0.8) mmol/l) than CAD patients (total cholesterol: 4.1 (0.8) mmol/l, LDL-C: 2.2 (0.5) mmol/l). Antidiabetic drugs were taken by 21.7% of the CAD patients, with mean HbA1c values in the prediabetic range (6.0 ± 0.7%). Twenty-four (52.2%) CAD patients had an eGFR below 60 ml/min/1.72 m^2^, while 15 (12.5%) apparently healthy individuals showed a mildly or moderately impaired eGFR. Triple-vessel coronary artery disease was present in 39.1% of CAD patients (vs. 28.3% for single- and 26.1% for double-vessel coronary artery disease). The number of affected coronary arteries was missing in the medical report for three patients. Preserved LVEF was found in 47.8% of CAD patients (vs. 26.1% for mildly reduced and 26.1% for reduced LVEF). Finally, the mean fasting time before blood sampling was at least 6.7 (3.2) hours in each group.

**Table 1:** Characteristics of patients with coronary artery disease and age- and sex-coarsened-matched healthy individuals.

	Healthy individuals		CAD patients	
	Females	Males	Females	Males
Participants, n (%)	38 (22.9)	82 (49.4)	4 (2.4)	42 (25.3)
Anthropometry, mean (SD)				
Age (years)	73.3 (4.6)	64.9 (15.9)	67.3 (12.2)	65.5 (14.3)
Body fat mass (%)	29.5 (7.4)	25.7 (7.4)	39.8 (2.2)	28.6 (5.3)
Body mass index (kg/m^2^)	23.6 (3.3)	23.6 (2.7)	27.8 (5.0)	27.6 (3.6)
Systolic blood pressure (mmHg)	126 (14)	128 (14)	139 (17)	127 (15)
Diastolic blood pressure (mmHg)	78 (11)	78 (9)	82 (16)	78 (10)
Cardiorespiratory fitness and PA levels, mean (SD)				
VO_2_peak (mL/min/kg)	24.8 (4.5)	31.7 (9.7)	16.3 (2.8)	22.7 (6.3)
Daily total PA (min/day)	253.2 (88.5)	259.0 (77.0)	261.5 (76.6)	206.1 (92.1)
Daily moderate-to-vigorous PA (min/day)	140.9 (63.9)	156.1 (58.0)	136.2 (36.8)	117.6 (65.8)
Smoking status, n (%)				
Never smoked	26 (15.7)	54 (32.5)	3 (1.8)	17 (10.2)
Ex-smokers (quit > 10 years ago)	12 (7.2)	28 (16.9)	1 (0.6)	7 (4.2)
Ex-smokers (quit < 10 years ago)	0 (0)	0 (0)	0 (0)	9 (5.4)
Active smokers	0 (0)	0 (0)	0 (0)	6 (3.6)
Missing information	0 (0)	0 (0)	0 (0)	3 (1.8)
Biochemical parameters, mean (SD)				
Fasting time prior to blood sampling (h)	6.6 (2.7)	6.8 (3.4)	8.8 (5.7)	8.1 (5.1)
Total cholesterol (mmol/L)	5.8 (0.9)	5.8 (1.2)	4.4 (0.5)	4.1 (0.9)
LDL-C (mmol/L)	3.3 (0.6)	3.2 (0.8)	2.0 (0.7)	2.2 (0.5)
HDL-C (mmol/L)	1.8 (0.4)	1.8 (0.6)	1.6 (0.7)	1.3 (0.2)
Triglycerides (mmol/L)	1.2 (0.6)	1.3 (0.6)	2.7 (2.0)	1.5 (0.8)
HbA1c (%)	5.4 (0.3)	5.3 (0.4)	5.6 (0.4)	6.0 (0.7)
Number of affected coronary arteries, n (%)				
None	38 (22.9)	82 (49.4)	0 (0)	0 (0)
Single-vessel	0 (0)	0 (0)	1 (0.6)	12 (7.2)
Double-vessel	0 (0)	0 (0)	0 (0)	12 (7.2)
Triple-vessel	0 (0)	0 (0)	3 (1.8)	15 (9.0)
Missing information	0 (0)	0 (0)	0 (0)	3 (1.8)
Left ventricular ejection fraction, n (%)				
Normal	38 (22.9)	82 (49.4)	0 (0)	0 (0)
Preserved (≥ 50%)	0 (0)	0 (0)	2 (1.2)	20 (12.0)
Mildly reduced (41–49%)	0 (0)	0 (0)	1 (0.6)	11 (6.6)
Reduced (≤ 40%)	0 (0)	0 (0)	1 (0.6)	11 (6.6)
Cardiometabolic medications, n (%)				
Anticoagulants or antiplatelets	0 (0)	0 (0)	4 (2.3)	42 (25.3)
Low-dose Aspirin	0 (0)	0 (0)	4 (2.3)	36 (21.7)
Antihypertensives	6 (3.4)	19 (10.9)	4 (2.3)	42 (25.3)
ACEI/ARB	6 (3.4)	16 (9.2)	3 (1.7)	37 (22.3)
Calcium antagonist	1 (0.6)	5 (2.9)	0 (0)	7 (4.2)
Betablocker	1 (0.6)	3 (1.7)	3 (1.7)	36 (21.7)
Lipid-lowering agents	3 (1.7)	6 (3.4)	4 (2.3)	41 (24.7)
Statins	3 (1.7)	6 (3.4)	4 (2.3)	40 (24.1)
Antidiabetic drugs	0 (0)	0 (0)	0 (0)	10 (6.0)
Oral antidiabetic drugs	0 (0)	0 (0)	0 (0)	8 (4.8)
Insulins	0 (0)	0 (0)	0 (0)	5 (3.0)
Estimated glomerular filtration rate categories, n (%)				
G1: normal or high	15 (39.5%)	13 (15.9%)	0 (0%)	3 (7.1%)
G2: mildly decreased	19 (50.0%)	58 (70.7%)	3 (75.0%)	16 (38.1%)
G3a: mildly to moderately decreased	3 (7.9%)	9 (11.0%)	1 (25.0%)	17 (40.5%)
G3b: moderately to severely decreased	1 (2.6%)	2 (2.4%)	0 (0%)	3 (7.1%)
G4: severely decreased	0 (0%)	0 (0%)	0 (0%)	2 (4.8%)
G5: kidney failure	0 (0%)	0 (0%)	0 (0%)	1 (2.4%)

*daily total PA* daily total physical activity, daily moderate-to-vigorous, *PA (min/day)* daily moderate-to-vigorous physical activity, *LDL-C* low-density lipoprotein cholesterol, *HDL-C* high-density lipoprotein cholesterol, *HbA1c* glycated haemoglobin, *ACEI* angiotensin converting enzyme inhibitors, *ARB* angiotensin receptor blocker.

### Associations between circulating amino acid levels and the presence of CAD

**Fig. 1:**
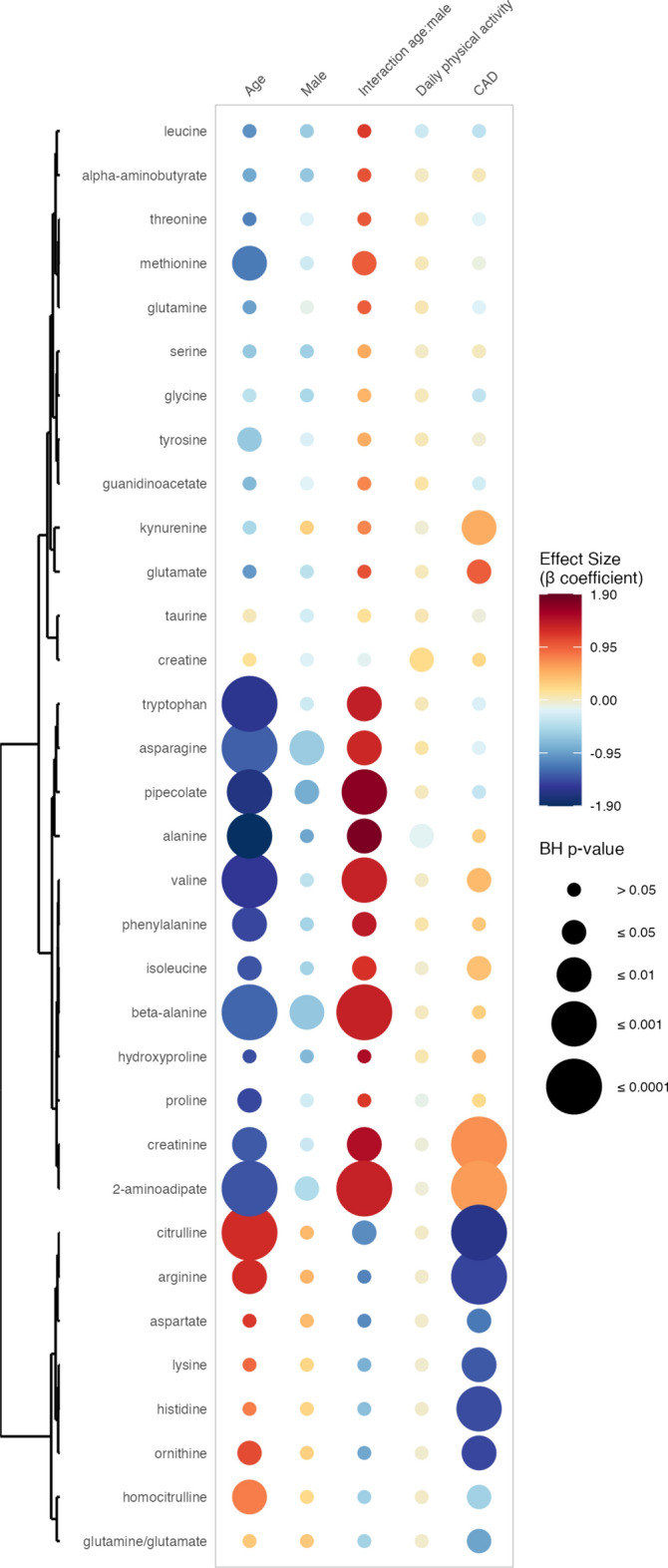
Associations between circulating amino acid levels and the presence of coronary artery disease. This rainplot represents the results of the first set of regression, in which metabolites were used as dependent variables (vertical axis), while CAD phenotype (two-level variable opposing sickness vs health) and confounders served as independent variables (horizontal axis). The redder the dots, the higher the β coefficient and the bigger the dot, the smaller the adjusted p-value. Metabolites with similar β coefficients and adjusted p-values were clustered together (left side of the rainplot). *BH* Benjamini–Hochberg.

Multiple amino acids displayed significantly different levels in patients with CAD compared to healthy participants, as displayed in Fig. 1. Specifically, amino acids implicated in the urea cycle, i.e., ornithine (β coefficient − 1.47, 95% CI [− 2.29, − 0.64], BH p-value ≤ 0.01), citrulline (β coefficient − 1.64 [− 2.28, − 1.00], BH p-value ≤ 0.0001), homocitrulline (β coefficient − 0.58 [− 0.99, − 0.17], BH p-value ≤ 0.05), aspartate (β coefficient − 1.18 [− 1.98, − 0.39], BH p-value ≤ 0.05) and arginine (β coefficient − 1.48 [− 2.09, − 0.87], BH p-value ≤ 0.0001), were all significantly and negatively associated with CAD. Glutamate (β coefficient 0.96 [0.32, 1.61], BH p-value ≤ 0.05), a precursor of ornithine feeding into the urea cycle, was significantly and positively associated with CAD. Although glutamine was not significantly different between CAD patients and healthy controls, the glutamine/glutamate ratio showed a significant and negative association with CAD (β coefficient − 0.92 [− 1.63, − 0.21], BH p-value ≤ 0.05). Additionally, lysine (β coefficient − 1.36 [− 2.07, − 0.65], BH p-value ≤ 0.01) and histidine (β coefficient − 1.43 [− 2.05, − 0.82], BH p-value ≤ 0.001) were significantly and negatively associated with CAD. Lastly, creatinine (β coefficient 0.68 [0.42, 0.93], BH p-value ≤ 0.0001), 2-aminoadipate (β coefficient 0.62 [0.42, 0.82], BH p-value ≤ 0.0001), kynurenine (β coefficient 0.52 [0.22, 0.82], BH p-value 0.01), valine (β coefficient 0.44 [0.16, 0.71], BH p-value 0.05) and isoleucine (β coefficient 0.39 [0.09, 0.69], BH p-value 0.05) were significantly and positively associated with CAD. All the results are available in the Supplementary Table 2.

### Association between circulating amino acid levels and the number of affected coronary arteries

**Fig. 2:**
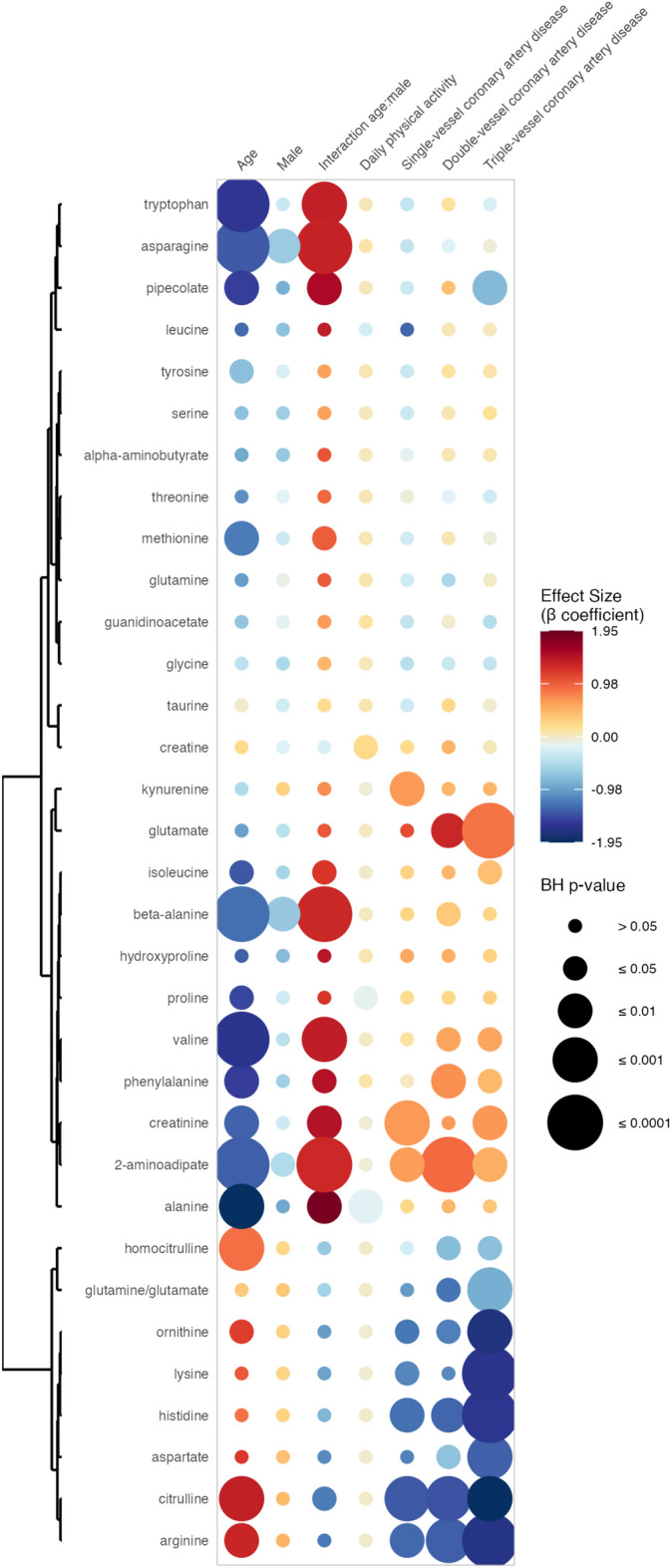
Associations between circulating amino acid levels and the number of affected coronary arteries. This rainplot represents the results of the second set of regression, in which metabolites were used as dependent variables (vertical axis). The number of affected coronary arteries (0, 1, 2 or 3) and confounders were independent variables (horizontal axis). The redder the dots, the higher the β coefficient and the bigger the dot, the smaller the adjusted p-value. Metabolites with similar β coefficients and adjusted p-values were clustered together (left side of the rainplot). *BH* Benjamini–Hochberg.

As shown in Fig. 2, the amino acids of the urea cycle (ornithine, citrulline, homocitrulline, aspartate, and arginine), as well as lysine and histidine, were also significantly and negatively associated with the number of affected coronary arteries. Importantly, the β coefficient tended to progressively decrease with the number of affected coronary arteries. Whereas glutamate was significantly and positively associated with double- and triple-vessel CAD, the glutamine/glutamate ratio showed a significant negative association with double- and triple-vessel CAD. Lastly, 2-aminoadipate was positively associated with single-, double- and triple-vessel CAD, while creatinine was positively associated with single- and triple-vessel CAD. Phenylalanine and valine were positively associated with double- and triple-vessel CAD. Kynurenine was positively associated with single-vessel CAD, β-alanine was positively associated with double-vessel CAD, isoleucine was positively associated with triple-vessel CAD, and pipecolate was negatively associated with triple-vessel CAD. All the detailed results are available in Fig. 2, and the Supplementary Table 3. β coefficients, 95% CI and BH p-values are intentionally omitted in this section to facilitate readability.

### Association between circulating amino acid levels and LVEF

**Fig. 3:**
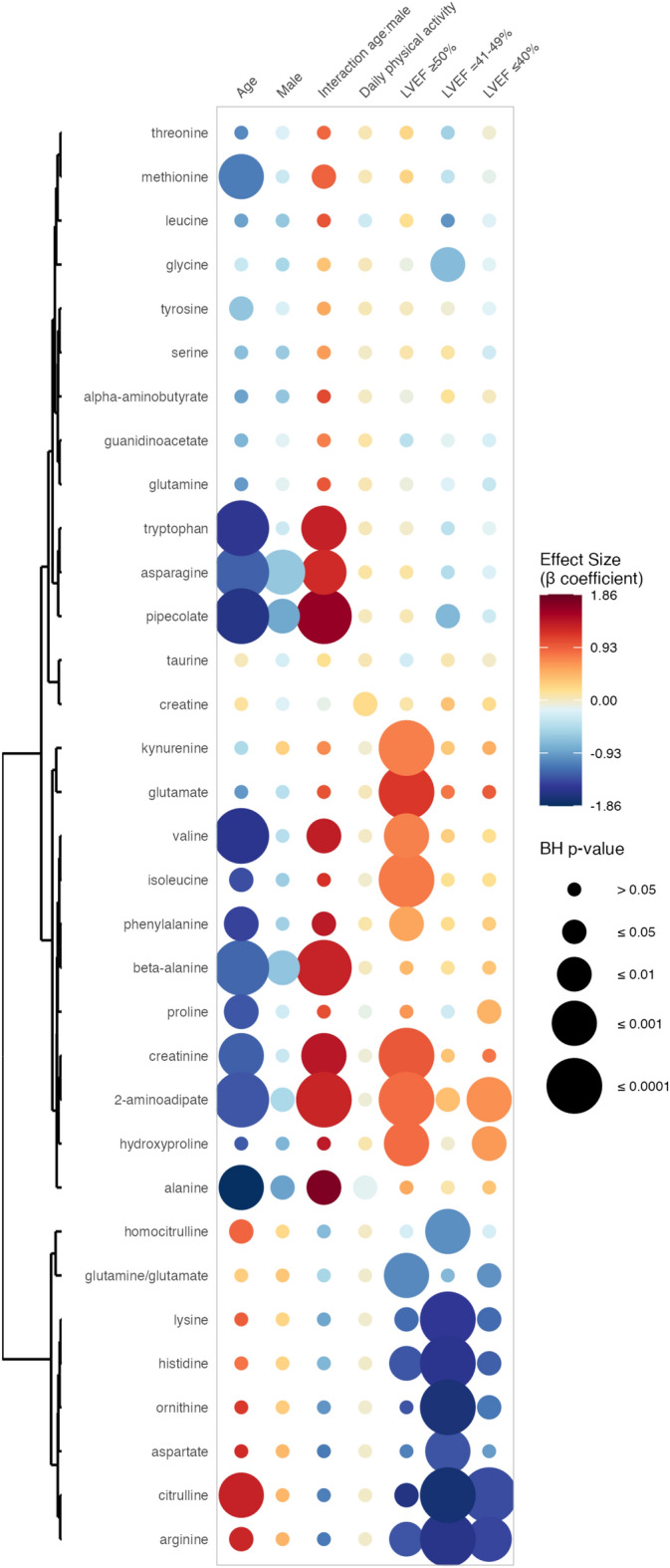
Associations between circulating amino acid levels and left ventricular ejection fraction impairment. This rainplot represents the results of the second set of regression, in which metabolites were used as dependent variables (vertical axis), while the level of left ventricular ejection fraction impairment (normal: healthy controls, preserved: LVEF ≥ 50%, mildly reduced: LVEF = 41–49%, and reduced: LEVF ≤ 40%). and confounders served as independent variables (horizontal axis). The redder the dots, the higher the β coefficient and the bigger the dot, the smaller the adjusted p-value. Metabolites with similar β coefficients and adjusted p-values were clustered together (left side of the rainplot). *BH* Benjamini–Hochberg, *LVEF* left ventricular ejection fraction.

Ornithine, citrulline, homocitrulline, aspartate, arginine, lysine, and histidine were significantly and negatively associated with LVEF impairment (Fig. 3). 2-aminoadipate was significantly and positively associated with all grades of LVEF impairment. The glutamine-to-glutamate ratio was significantly and negatively associated with preserved and reduced LVEF. Hydroxyproline was significantly and positively associated with preserved and reduced LVEF, while proline was significantly and positively associated with reduced LVEF only. Glutamate, kynurenine, creatinine, valine, isoleucine, and phenylalanine were significantly and positively associated with preserved LVEF (≥ 50%). Finally, glycine and pipecolate were significantly and negatively associated with mildly reduced LVEF. All the detailed results are available in Fig. 3, and the Supplementary Table 4. β coefficients, 95% CI and BH p-values are intentionally omitted in this section to facilitate readability.

### Association between circulating amino acids and covariables

Four of the five urea cycle amino acids (citrulline, homocitrulline, arginine, and ornithine) were significantly and positively associated with age in the three sets of multiple linear regressions. Aspartate was the only urea cycle amino acid which was not significantly associated with age. With the exception of citrulline, none of these amino acids was significantly associated with the interaction term age:male, which means that the associations observed between amino acids and age are valid for both females and males. None of the urea cycle amino acids showed any association with the male sex. Lysine, histidine, glutamate, the glutamine/glutamate ratio and kynurenine did not show any significant association with age, male or the interaction term age:male.

Methionine, tryptophan, asparagine, pipecolate, alanine, valine, phenylalanine, isoleucine, beta-alanine, proline, creatinine, and 2-aminoadipate were significantly and negatively associated with age but significantly and positively associated with the interaction term age:male. Asparagine, pipecolate, beta-alanine, and 2-aminoadipate were also significantly and negatively associated with the male sex. Finally, creatine was significantly and positively associated with daily physical activity, while alanine was significantly and negatively associated with daily physical activity. All the detailed results are available in the Supplementary Tables 1 to 3. β coefficients, 95% CI and BH p-values are intentionally omitted in this section to facilitate readability.

### Alternative models adjusted for the estimated glomerular filtration rates

In these alternative models, 24 CAD patients (52.2%) and 15 apparently healthy individuals (12.5%) were excluded for having an eGFR below 60 ml/min/1.72 m^2^, leaving the total of included participants to 105 healthy participants (67.7% male) and 22 age- and sex-coarsened-matched CAD patients (86.3% male). The results obtained with these alternatives models are similar to the results obtained with the primary models. In particular, the circulating levels of the urea cycle amino acids (ornithine, citrulline, homocitrulline, aspartate, and arginine) remained negatively associated with the presence of CAD (Supplementary Fig. 2 and Supplementary Table 5), with the number of affected coronary arteries (Supplementary Fig. 3 and Supplementary Table 6) and with LVEF (Supplementary Fig. 4 and Supplementary Table 7).

## Discussion

**Fig. 4:**
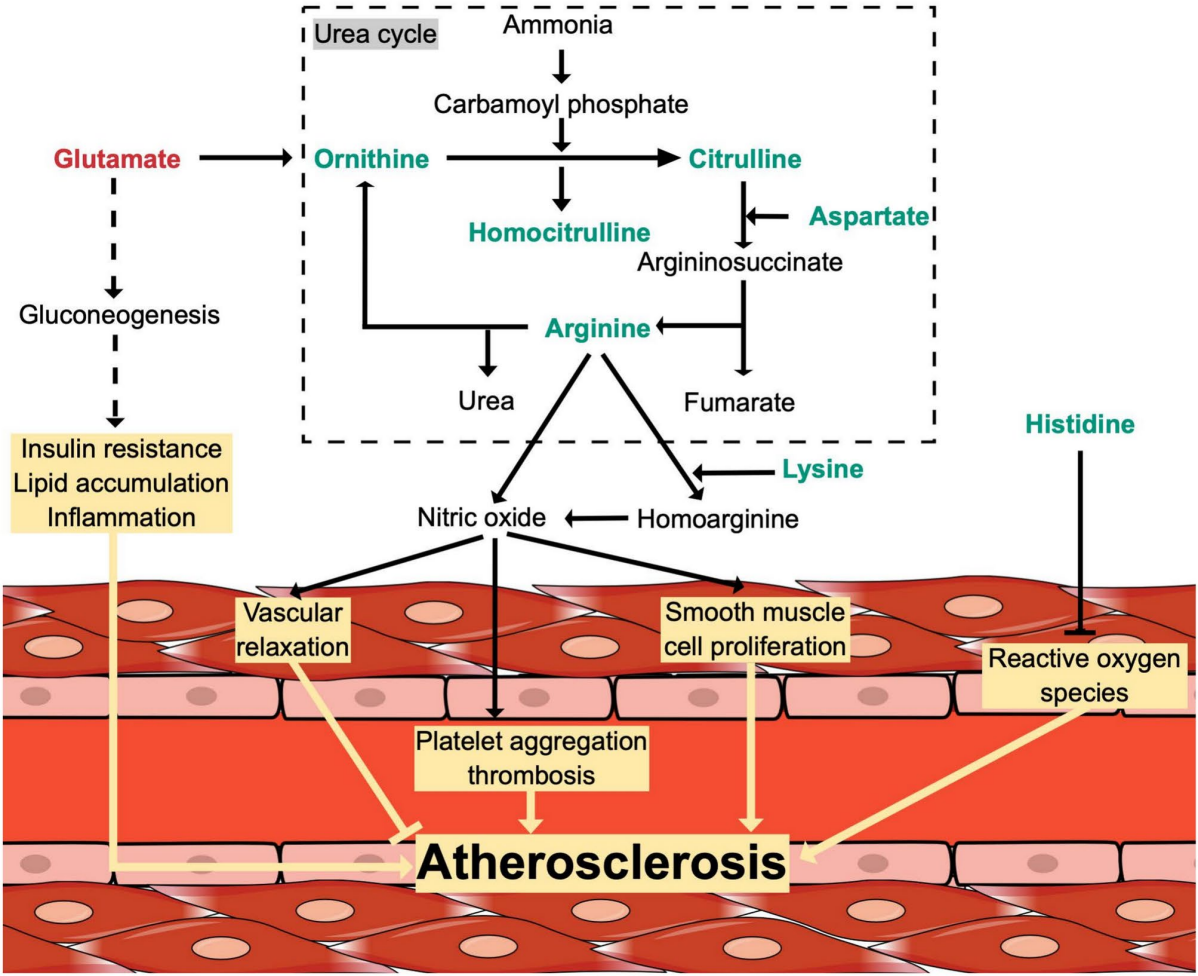
(Graphical abstract): Findings of the present study illustrated in their biological context. The urea cycle occurs in the liver and, to a lesser extent, in the kidneys. Amino acid catabolism produces ammonia, the accumulation of which is toxic to the body. The urea cycle aims to detoxify ammonia to urea, which is then excreted by the kidneys. The conversion of ammonia to carbamoyl phosphate, as well as the conversion of ornithine and carbamoyl phosphate to citrulline, take place in mitochondria. The other reactions of the urea cycle happen in the cytosol. In addition to producing urea, the urea cycle is an important source of nitric oxide, which is key to maintaining endothelial homeostasis. The nitric oxide-producing amino acids were negatively associated with coronary artery disease in the present study, which might reflect the overconsumption of these amino acids to compensate for the increased nitric oxide demand in patients with coronary artery disease. By driving gluconeogenesis, glutamate is thought to promote lipid accumulation, insulin resistance and inflammation. Histidine can buffer reactive oxygen species. Amino acids highlighted in green were found to be negatively associated with coronary artery disease, while amino acids highlighted in red were positively associated with coronary artery disease. Molecules and pathways written in black are closely related to the urea cycle but were not quantified in the present study. Yellow boxes indicate physiological and pathophysiological consequences. Dashed arrows indicate potential pathways, as hypothesised by Zheng et al.^76^. The figure is based on^37,77–79^ and was drawn using https://www.mindthegraph.com (accessed on 08.02.23).

As summarised in Fig. 4, the present study showed evidence that circulating levels of the urea cycle amino acids (ornithine, citrulline, homocitrulline, aspartate, and arginine) were negatively associated with CAD. There was also evidence that lysine, histidine, and the glutamine/glutamate ratio were negatively associated with CAD. Kynurenine, glutamate, valine, isoleucine, 2-aminoadipate and creatinine were positively associated with the presence of CAD. These findings remain valid regardless of the independent variable used to represent CAD phenotypes (a binary variable CAD vs. healthy, the number of affected coronary arteries, or the LVEF), highlighting their robustness.

### Urea cycle amino acids feature the impaired endothelial function associated with CAD

The urea cycle mainly occurs in the liver and has two critical roles in the human body^36^. First, it aims to detoxify ammonia, a product of amino acid catabolism, into harmless urea, which is then excreted by the kidneys^37^. Second, it produces nitric oxide, which is essential to the endothelial function of blood vessels^37^. Nitric oxide relaxes the smooth vasculature and dilates blood vessels^37^. This relaxation is impaired in patients with CAD due to reduced nitric oxide production and increased inactivation of nitric oxide^37^. As the nitric oxide-producing amino acids were negatively associated with CAD phenotypes, this might reflect the overconsumption of these amino acids to compensate for the increased nitric oxide demand.

Arginine levels were lower in patients with CAD compared to healthy controls. Through a transformation catalyzed by the nitric oxide synthetase, arginine is a precursor of nitric oxide and is, therefore, involved in regulating endothelial function^38^. Even though the clinical relevance of arginine as a limiting factor for the bioavailability of nitric oxide remains a matter of debate^39,40^. Low arginine levels have been previously associated with atherosclerosis, which aligns with the present findings^41^. Lysine, which also displayed lower levels in CAD patients, is another nitric oxide source. Indeed, the arginine-glycine amidino-transferase converts lysine to homoarginine, which the nitric oxide synthetase can then metabolize to produce nitric oxide^42^. As the nitric oxide synthetase holds a lower affinity for homoarginine than arginine, the observed lower lysine levels in patients with CAD might indicate an important nitric oxide demand. In addition, inflammation states, such as CAD, promote the carbamylation of lysine to protein-bound homocitrulline^43^. Carbamylation, a non-enzymatic binding of isocyanic, lowers circulating lysine and increases protein-bound homocitrulline levels in CAD patients^44^. While circulating homocitrulline was lower in patients with CAD, it must be pointed out that free and not protein-bound homocitrulline was measured. Therefore, a shift from free homocitrulline to protein-bound homocitrulline could occur in patients with CAD.

### Glutamate under the spotlight

Glutamate was positively associated with CAD. This finding aligns with previous reports showing elevated glutamate levels in subjects with cardiovascular risk factors and diseases^45,46^. By driving gluconeogenesis, glutamate is thought to promote traditional cardiovascular risk factors such as obesity and insulin resistance^47,48^. There is evidence that circulating glutamate might also have pro-inflammatory effects^49^. As nutrition is a significant source of glutamate in humans (primarily through the consumption of animal proteins, including red meats, poultry, fish, and dairy products), it is believed that an elevated glutamate intake could favour the clustering of traditional cardiovascular risk factors and eventually the development of CAD^50^.

While elevated glutamine level was previously linked to lower cardiovascular risk^46^, no significant association between glutamine and CAD phenotypes was found in the present study. Therefore, the fact that the glutamine/glutamate ratio was found to be significantly and negatively associated with CAD is due to the elevated glutamate levels observed in patients with CAD. Nonetheless, glutamine is considered to be involved, as an indirect arginine precursor, in nitric oxide synthesis and the production of the anti-oxidative glutathione^51^.

### Circulating histidine, a potential oxidative stress scavenger

The involvement of oxidative stress in the pathophysiology of CAD is well-recognized^52^. Elevated levels of reactive oxygen species inflict oxidative damage to macromolecules, impair vascular physiology, and ultimately promote CAD development^52^. Radical scavengers such as histidine can buffer reactive oxygen species^53^. Furthermore, histidine and alanine can form carnosine, another radical scavenger, especially in muscle tissue^54^. In that way, lower histidine levels in patients with CAD might sign an increased consumption of histidine to scavenge reactive oxygen species^54^. Lastly, in heart failure patients, in which oxidative stress also plays a pivotal role, histidine is negatively associated with B-type natriuretic peptide (BNP) but positively associated with serum albumin concentration, which might indicate that skeletal muscle wasting could also partly explain lower circulating histidine levels in cardiovascular diseases^55^.

### Kynurenine, 2-aminoadipate, creatinine, valine and isoleucine in the context of CAD

Kynurenine is a product of tryptophan catabolism, mainly occurring in the liver^56^. Kynurenine and related metabolites are involved in maintaining vascular immune homeostasis^57^. Alterations in their metabolism have been reported in the pathogenesis of atherosclerosis^56^. Specifically, elevated blood kynurenine levels were shown to predict death and recurrent myocardial infarction in patients with CAD^56,58^ and acute myocardial infarction in patients with suspected stable angina pectoris^59^. Kynurenine levels were also associated with cardiovascular disease prevalence in end-stage renal disease patients^60^. These findings align with the present observation that CAD patients displayed higher kynurenine levels than healthy controls. Overall, the kynurenine pathway seems to be a promising research area regarding CAD pathophysiology.

2-Aminoadipate, a product of lysine catabolism, displayed elevated levels in patients with CAD compared to healthy controls in the present study^61^. Higher 2-aminoadipate levels have been associated with type 2 diabetes^62^, insulin resistance^61^, and coronary artery calcification^63^. Recently, elevated levels of 2-aminoadipate were linked to reduced high-density lipoprotein cholesterol and elevated triglyceride levels^64,65^, highlighting a potential mechanism through which elevated 2-aminoadipate could negatively influence cardiometabolic health.

Creatine is produced primarily in the liver from the methylation of guanidinoacetate and synthesized in kidneys from the amino acids arginine and glycine^66^. In skeletal muscle, creatine conversion to phosphocreatine forms creatinine, which diffuses from the cells and is excreted into the urine by glomerular filtration and partial tubular excretion^66^. The latter explains that creatinine is a classical marker of kidney function in clinical medicine^67^. Even though creatinine is considered to be a biologically inactive metabolite^66^, elevated creatinine levels have been associated with increased mortality in CAD patients^68^. Interestingly, plasma creatinine levels were related to arterial stiffness in untreated hypertensive subjects with preserved renal function^69^. Whether creatinine plays a biologically active role in atherosclerosis remains unknown and will require further investigation.

Valine and isoleucine belong to the branched-chain amino acids (BCAA). In humans, diet is the first provider of BCAA, even though a small part is synthesized by the gut microbiome^10^. Elevated levels of circulating BCAA have been observed in several cardiometabolic diseases, including atherosclerosis and CAD^10^. Interestingly, elevated circulating BCAA levels have been shown to be predictive of CAD severity^70,71^. This was also found in the present study, as there was evidence for positive associations with double- and triple-vessel CAD (valine) or even with triple-vessel CAD only (isoleucine). Molecular mechanisms linking BCAA and atherosclerosis or CAD remain largely unknown but could involve activation of the serine/threonine protein kinase mTOR, which leads to more reactive oxygen species production and endothelial dysfunction^72^, or activation of platelet-dependent pro-thrombotic pathways^73^. In the present study, there was no evidence that leucine, the third BCAA, was associated with any CAD phenotype.

### Amino acids—key players in the inflammatory progression of atherosclerosis?

Atherosclerosis is now recognized as a chronic inflammatory disease of the arterial wall driven by lipids and immune cells^11^. The CANTOS trial, which used canakinumab, a therapeutic monoclonal antibody, to block interleukin-1β, proved that targeting the immune system effectively reduces cardiovascular events^74^. As amino acids are highly involved in the immune regulation of endothelial, innate and adaptive immune cells, they could become future therapeutic targets^11^. Therefore, understanding amino acid metabolism in atherosclerosis is worthy of interest. For instance, pro- and anti-inflammatory cytokines act differently on the arginine pathway, promoting macrophage conversion into proinflammatory M1 or anti-inflammatory M2 types^11^. Nevertheless, the mechanisms through which amino acids influence the pathogenesis of atherosclerosis are largely unknown and require further research.

### Limitations and strengths

This study should be assessed considering its limitations. First, the cross-sectional nature of this study allows only for the investigation of associations, and not causality, between amino acids and CAD phenotypes. Second, investigating circulating metabolites does not reveal their cellular origin or destination. Thus, the present findings regarding hypothetical molecular mechanisms of action should be interpreted cautiously. Third, the small number of patients with triple-vessel CAD or LVEF ≤ 40% limits the extrapolation of the present results to severely sick patients. Fourth, as more than 90% of the included CAD patients were male, it is unclear to what extent the reported amino acid signature applies to female CAD patients. Finally, the findings reported in this manuscript should be replicated in larger population studies before any translation to clinical practice can be attempted.

It has been demonstrated that food intake and fasting only have minimal impact on circulating amino acid levels^75^. Nevertheless, serum samples were collected in a fasting state of at least 6.5 h on average, and regression analyses were adjusted for fasting time, further enhancing the robustness of the present findings. Overall, this cross-sectional study should be seen as an incentive to investigate amino acids further in the context of CAD pathophysiology and cardiovascular risk stratification.

## Conclusion

The amino acids of the urea cycle, the antioxidant histidine and lysine, were negatively associated with CAD phenotypes, while glutamate was positively associated with them. We hypothesise that this circulating amino acid signature could feature pathophysiological changes commonly observed in CAD, including greater nitric oxide consumption, impaired endothelial function, and elevated oxidative stress. Overall, amino acid profiling seems a promising pathophysiology-based phenotyping tool to stratify cardiovascular risk. However, replication of the present findings in larger-scale and prospective population studies is required before amino acid profiling can be translated to clinical practice.

## Supplementary Information

Supplementary Figures. The file containing the supplementary Figures is still is track change mode. Please convert it into a clean version. If you are unsure if it is fine or not, please send a second proof to review. Thank you.

Supplementary Table 1.

Supplementary Table 2.

Supplementary Table 3.

Supplementary Table 4.

Supplementary Table 5.

Supplementary Table 6.

Supplementary Table 7.

## Data Availability

All data are available as supplements to the present manuscript.
