# Supplementary material for: Circulating amino acid signature features urea cycle alterations associated with coronary artery disease
Source: Sci Rep. 2024 Oct 28;14:25848. doi: 10.1038/s41598-024-76835-7 (PMC11519371; doi:10.1038/s41598-024-76835-7)
Supplement: Supplementary file 1 — Supplementary Figures. The file containing the supplementary Figures is still is track change mode. Please convert it into a clean version. If you are unsure if it is fine or not, please send a second proof to review. Thank you. [file 41598_2024_76835_MOESM1_ESM.docx]

**Supplementary figures**

***Supplementary Figure 1: Directed acyclic graph showing the relationships between amino acids and coronary artery disease***

*To determine for which variables regressions needed to be adjusted, a directed acyclic graph (DAG) was drawn (1). Coronary artery disease was defined as the exposure and amino acids as the outcome. The blue circles are the ancestors of outcome, and the pink circles are the ancestors of both exposure and outcome. The green line is the causal path, and the pink lines are the biasing path. Age, sex, and daily physical activity were identified as variables for which regressions should be adjusted (2). Sampling time and fasting time were identified as variables to be included in the linear mixed models to reduce the outcome variation and improve the precision of the average effect of CAD on amino acids (3, 4). Abbreviations: CAD: Coronary artery disease. This figure was created using* [*http://www.dagitty.net*](http://www.dagitty.net) (accessed on 04.04.22)]*.(1)*


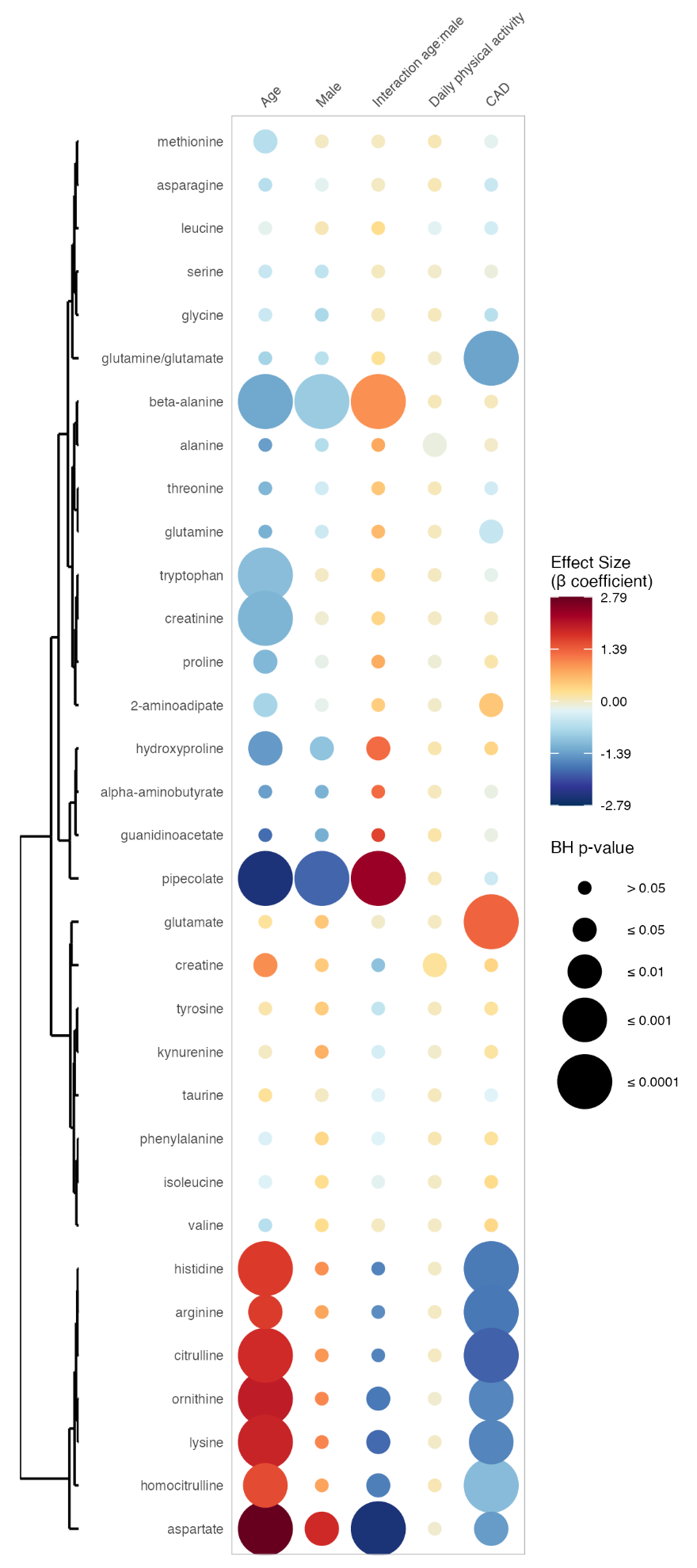


***Supplementary Figure 2: Associations between circulating amino acid levels and the presence of coronary artery disease, adjusted for the estimated glomerular filtration rate***

*This rainplot represents the results of the alternative model to the first set of regression, in which metabolites were used as dependent variables (vertical axis), while CAD phenotype (two-level variable opposing sickness vs health) and confounders served as independent variables (horizontal axis). In this alternative model, participants with kidney failure (defined as an estimated glomerular filtration rate, abbreviated eGFR, lower than 60 ml/min/1.73m^2^) were excluded, and eGFR was used as an additional variable for which regressions were adjusted* (5). *The redder the dots, the higher the β coefficient and the bigger the dot, the smaller the adjusted p-value. Metabolites with similar β coefficients and adjusted p-values were clustered together (left side of the rainplot). Abbreviations: BH = Benjamini-Hochberg*


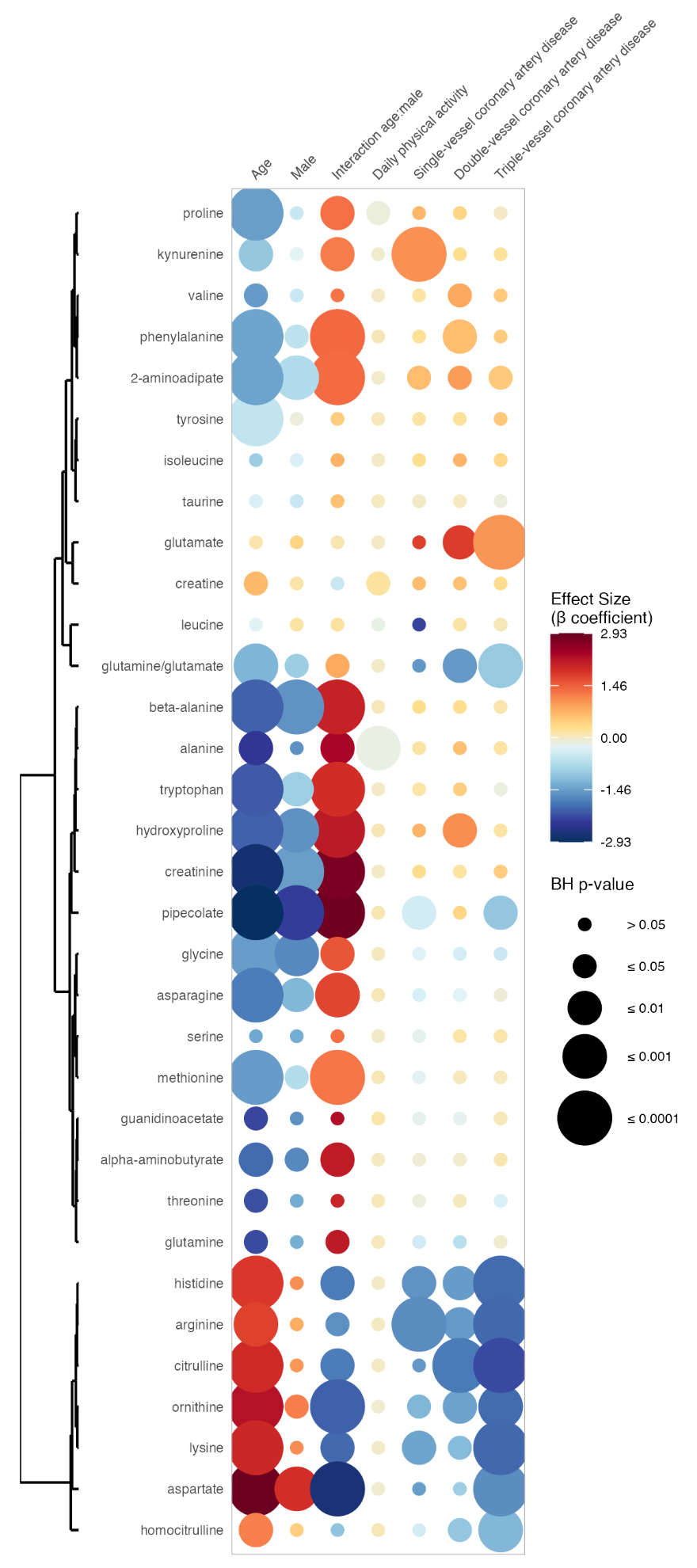


***Supplementary Figure 3: Associations between circulating amino acid levels and the number of affected coronary arteries, adjusted for the estimated glomerular filtration rate***

*This rainplot represents the results of the alternative model to the second set of regression, in which metabolites were used as dependent variables (vertical axis). The number of affected coronary arteries (0, 1, 2 or 3) and confounders were independent variables (horizontal axis). In this alternative model, participants with kidney failure (defined as an estimated glomerular filtration rate, abbreviated eGFR, lower than 60 ml/min/1.73m^2^) were excluded, and eGFR was used as an additional variable for which regressions were adjusted* (5). *The redder the dots, the higher the β coefficient and the bigger the dot, the smaller the adjusted p-value. Metabolites with similar β coefficients and adjusted p-values were clustered together (left side of the rainplot). Abbreviations: BH = Benjamini-Hochberg*


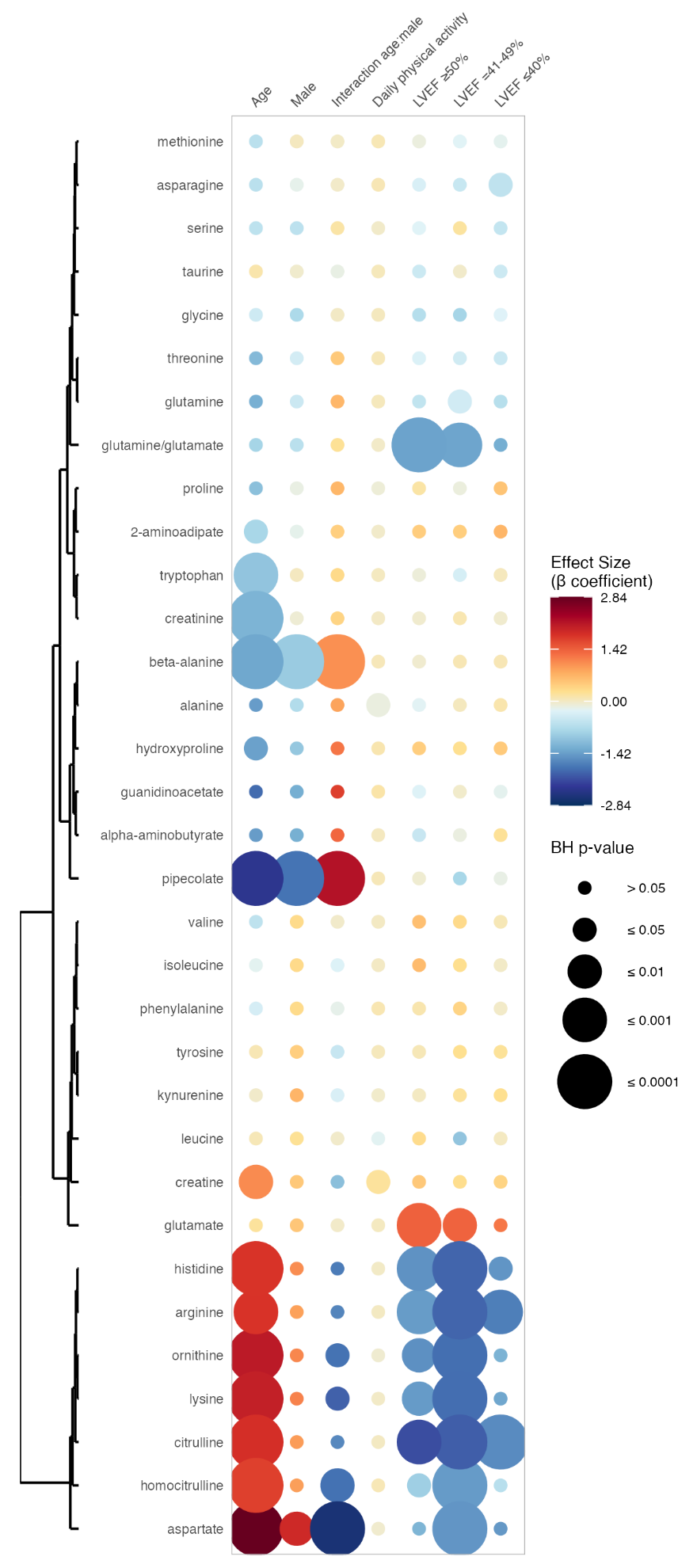


***Figure 3: Associations between circulating amino acid levels and left ventricular ejection fraction impairment, adjusted for the estimated glomerular filtration rate***

*This rainplot represents the results of the alternative model to the second set of regression, in which metabolites were used as dependent variables (vertical axis), while the level of left ventricular ejection fraction impairment (normal: healthy controls, preserved: LVEF ≥50%, mildly reduced: LVEF = 41-49%, and reduced: LEVF≤40%). and confounders served as independent variables (horizontal axis). In this alternative model, participants with kidney failure (defined as an estimated glomerular filtration rate, abbreviated eGFR, lower than 60 ml/min/1.73m^2^) were excluded, and eGFR was used as an additional variable for which regressions were adjusted* (5). *The redder the dots, the higher the β coefficient and the bigger the dot, the smaller the adjusted p-value. Metabolites with similar β coefficients and adjusted p-values were clustered together (left side of the rainplot). Abbreviations: BH = Benjamini-Hochberg, LVEF = Left ventricular ejection fraction.*

**References**

1. Textor J, van der Zander B, Gilthorpe MS, Liskiewicz M, Ellison GT. Robust causal inference using directed acyclic graphs: the R package 'dagitty'. Int J Epidemiol. 2016;45(6):1887-94.

2. Felder TK, Ring-Dimitriou S, Auer S, Soyal SM, Kedenko L, Rinnerthaler M, et al. Specific circulating phospholipids, acylcarnitines, amino acids and biogenic amines are aerobic exercise markers. J Sci Med Sport. 2017;20(7):700-5.

3. Tokarz J, Adamski J. Chapter 2 - Confounders in metabolomics. In: Adamski J, editor. Metabolomics for Biomedical Research: Academic Press; 2020. p. 17-32.

4. Ang JE, Revell V, Mann A, Mäntele S, Otway DT, Johnston JD, et al. Identification of Human Plasma Metabolites Exhibiting Time-of-Day Variation Using an Untargeted Liquid Chromatography–Mass Spectrometry Metabolomic Approach. Chronobiology International. 2012;29(7):868-81.

5. Stevens PE, Ahmed SB, Carrero JJ, Foster B, Francis A, Hall RK, et al. KDIGO 2024 Clinical Practice Guideline for the Evaluation and Management of Chronic Kidney Disease. Kidney International. 2024;105(4):S117-S314.
